# Ethyl 4-(3-benzoyl­thio­ureido)benzoate

**DOI:** 10.1107/S1600536808017856

**Published:** 2008-07-12

**Authors:** Sohail Saeed, Moazzam Hussain Bhatti, Uzma Yunus, Peter G. Jones

**Affiliations:** a Department of Chemistry, Allama Iqbal Open University, Islamabad, Pakistan; b Institut für Anorganische und Analytische Chemie, Technische Universität Braunschweig, Postfach 3329, 38023 Braunschweig, Germany

## Abstract

The title compound, C_17_H_16_N_2_O_3_S, crystallizes in the thio­amide form with an intra­molecular N—H⋯O hydrogen bond across the thio­urea system. Mol­ecules are connected in chains parallel to [10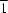] by hydrogen bonds from the second thio­urea N—H group to the benzoate C=O function.

## Related literature

For related literature, see: Huebner *et al.* (1953 ▶); Xu *et al.* (2004 ▶); Xue *et al.* (2003 ▶); Zeng *et al.* (2003 ▶); Zheng *et al.* (2004 ▶); Douglas & Dains (1934 ▶); Glasser & Doughty (1964 ▶); Morales *et al.* (2000 ▶); D’hooghe *et al.* (2005 ▶); Dušek (1985 ▶).
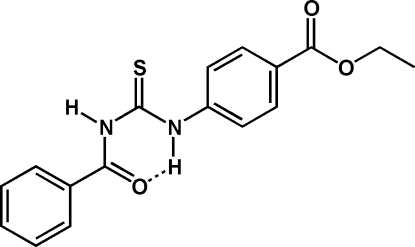


## Experimental

### 

#### Crystal data

C_17_H_16_N_2_O_3_S
*M* *_r_* = 328.38
Monoclinic, 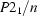

*a* = 9.6018 (3) Å
*b* = 8.3882 (3) Å
*c* = 19.3199 (6) Å
β = 91.393 (4)°
*V* = 1555.60 (9) Å^3^
*Z* = 4
Mo *K*α radiation
μ = 0.23 mm^−1^
*T* = 100 (2) K
0.38 × 0.24 × 0.13 mm

#### Data collection

Oxford Diffraction Xcalibur S diffractometer
Absorption correction: multi-scan (*CrysAlis RED*; Oxford Diffraction, 2008 ▶) *T*_min_ = 0.943, *T*_max_ = 1.000 (expected range = 0.916–0.971)
31428 measured reflections
5103 independent reflections
3676 reflections with *I* > 2σ(*I*)
*R*_int_ = 0.048

#### Refinement

*R*[*F* ^2^ > 2σ(*F* ^2^)] = 0.037
*wR*(*F* ^2^) = 0.092
*S* = 0.94
5103 reflections
217 parameters
H atoms treated by a mixture of independent and constrained refinement
Δρ_max_ = 0.43 e Å^−3^
Δρ_min_ = −0.29 e Å^−3^

### 

Data collection: *CrysAlis RED* (Oxford Diffraction, 2008 ▶); cell refinement: *CrysAlis RED*; data reduction: *CrysAlis RED*; program(s) used to solve structure: *SHELXS97* (Sheldrick, 2008 ▶); program(s) used to refine structure: *SHELXL97* (Sheldrick, 2008 ▶); molecular graphics: *XP* (Siemens, 1994 ▶); software used to prepare material for publication: *SHELXL97*.

## Supplementary Material

Crystal structure: contains datablocks I, global. DOI: 10.1107/S1600536808017856/im2071sup1.cif

Structure factors: contains datablocks I. DOI: 10.1107/S1600536808017856/im2071Isup2.hkl

Additional supplementary materials:  crystallographic information; 3D view; checkCIF report

## Figures and Tables

**Table 1:** Hydrogen-bond geometry (Å, °)

*D*—H⋯*A*	*D*—H	H⋯*A*	*D*⋯*A*	*D*—H⋯*A*
N2—H02⋯O1	0.85 (2)	1.89 (2)	2.618 (1)	143 (1)
N1—H01⋯O2^i^	0.85 (2)	2.28 (2)	3.099 (1)	162 (1)
C3—H3⋯S^ii^	0.95	3.04	3.763 (1)	134
C17—H17*C*⋯S^iii^	0.98	2.88	3.677 (1)	140

Symmetry codes: (i) 
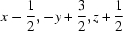
; (ii) 
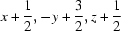
; (iii) 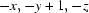.

**Fig. 1.**
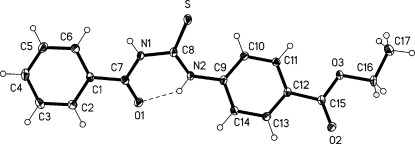
The molecule of the title compound in the crystal. Ellipsoids represent 50% probability levels.

**Fig. 2.**
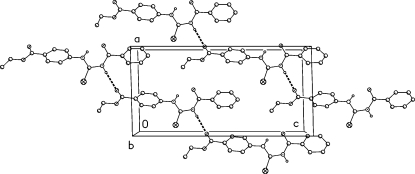
Packing diagram of I showing classical H bonds as thick dashed bonds. H atoms not involved in H bonds are omitted for clarity.

## supplementary crystallographic information

### Comment

Epoxy resins have the combination of good thermal and dimensional stability, excellent chemical and corrosion resistance, high tensile strength and modulus, and ease of handling and processability, ensuring their wide application in the aerospace and electronic industries in the form of structural adhesives, advanced composite matrices, and packaging materials (Dušek, 1985). The properties of cured epoxy polymers largely depend on the nature of the chemical structure of the starting resins and curing agents. The title compound (I) is a precursor in an attempt to synthesize imidazole derivatives and transition metal complexes as epoxy resin curing agents and accelerators. Substituted thioureas are an important class of compounds, precursors or intermediates towards the synthesis of a variety of heterocyclic systems such as imidazole-2-thiones (Zeng *et al.*, 2003), 2-imino-1, 3-thiazolines (D'hooghe *et al.*, 2005), pyrimidine-2-thiones and (benzothiazolyl)-4-quinazolinones. Thioureas are also known to exhibit a wide range of biological activities including antiviral, antibacterial, antifungal, antitubercular, antithyroidal, herbicidal and insecticidal activities (Huebner *et al.*, 1953) and as agrochemicals (Xu.Y *et al.*, 2004). One example are 1-benzoyl-3-(4,5-disubstituted-pyrimidine-2-yl) thioureas, which have excellent herbicidal activity (W.Zheng *et al.*, 2004). Thioureas are also well known chelating agents for transition metals (Xue *et al.*,2003). *N*,*N*-Dialkyl-*N*'-benzoyl thioureas act as selective complexing agents for the enrichment of platinum metals even from strongly interfacing matrixes.The complexes of thiourea derivatives also show various biological activities (Glasser *et al.*, 1964). Thioureas and substituted thioureas are also known as epoxy resin curing agents. We became interested in the synthesis of N-Aroyl, *N*'-arylthioureas as intermediates towards some new novel heterocycles and for the systematic study of their bioactive complexes and epoxy resin curing agents. In this article, we describe the spectroscopy and crystal structure of ethyl 4-(3-benzoylthioureido)-benzoate (I) as a typical representative of N-aroyl, *N*'-arylthioureas. Compound (I) crystallizes in the thioamide form. The conformation of the molecule with respect to the carbonyl and thiocarbonyl part is essentially planar, as reflected by the torsional angles O1—C7—N1—C8, C7—N1—C8—S and C7—N1—C8—N2 of 0.7 (2), -177.97 (9) and 1.0 (2) °, respectively. However, there is rotation about the various moieties as indicated by *e.g.* C6—C1—C7—N1 34.7 (2) and C8—N2—C9—C14 136.9 (1) °. Apart from the atoms O1, N1, C8 and S, the molecule is planar (mean deviation of non-H atoms is 0.055 Å). The C7—O1, C8—S and C15—O2 bonds show a typical double bond character with bond lengths of 1.226 (1), 1.659 (1) and 1.215 (1) Å, respectively. All of the C—N bonds, C9—N2 1.415 (1), C7—N1 1.385 (1), C8—N2 1.339 (2), and C8—N1 1.401 (1) Å also indicate partial double bond character. Among the three latter C—N bonds, C7—N1 is the longest, indicating an C(*sp*^2^)—N(*sp*^2^) single bond, while C8—N2 is the shortest bond with more double bond character. This demonstrates that there is π conjugation in the system S—C8—N2 but not along O1—C7—N1 and C7—N1—C8, as found in 1-(3-methoxybenzoyl)-3,3-diethylthiourea (Moraless *et al.*, 2000).

There is a strong intramolecular hydrogen bond N2—H02···O1, with distances H2···O1 1.89 (2) and N2···O1 2.618 (1) Å, resulting in a 6-membered ring. Molecules are connected in chains parallel to [101] by the classical H bond N1—H01···O2; weak C—H···S interactions are observed interconecting the chains (Table 1).

### Experimental

The title compound was synthesized by a slight modification of the published procedure (Douglas *et al.*, 1934). A solution of benzoyl chloride (0.1 mol) in anhydrous acetone (70 ml) was added dropwise to a suspension of ammonium thiocyanate (0.1 mol) in anhydrous acetone (50 ml) and the reaction mixture was refluxed for 45 minutes. After cooling to room temperature, a solution of *p*-aminobenzoic acid ethyl ester (0.1 mol) in anhydrous acetone (25 ml) was added and the resulting mixture refluxed for 1.5 hrs. The reaction mixture was poured into five times its volume of cold water where the thiourea precipitated as a solid. The product was recrystallized from ethyl acetate as pale yellow crystals (3.55 g, 85%). m.p. 425 K. Elemental analysis for C_17_H_16_N_2_O_3_S (*M*=328.38) calc. C 62.19, H 4.87, N 8.53, S 9.75, found C 62.16, H 4.93, N 8.58, S 9.76. FTIR (KBr pellet) [cm^-1^]: 1276 (C=S), 1676 (C=O amide), 1700 (C=O ester), 3346 (free N—H), 3208 (assoc. N—H). ^1^H-NMR (400 MHz, DMSO-d^6^) [ppm]: 1.34 (3*H*, t, CH_3_); 4.32 (2*H*, q, CH_2_); 7.51–7.56 (2*H*, m, CH_ar_), 7.63–7.68 (2*H*, m, CH_ar_), 7.90–8.00 (5*H*, m, CH_ar_); 11.63 (1*H*, s, broad, NH); 12.80 (1*H*, s, broad, NH). ^13^C-NMR (300 MHz, DMSO-d^6^) [ppm]: 14.14 (CH_3_); 60.70 (CH_2_); 127.83(C), 128.72(C), 128.81(C), 129.72(C), 132.06(C), 133.17(C); 165.08(C=O amide); 168.20 (C=O ester), 178.99 (C=S thioamide).

### Refinement

H atoms of NH groups were refined freely. Methyl H atoms were included on the basis of idealized rigid groups (C—H 0.98 Å, H—C—H 109.5°) allowed to rotate but not tip. Other hydrogen atoms were included using a riding model with C—H 0.95 (aromatic) or 0.99 (methylene) Å. U(H) values were fixed at 1.5*U*_iso_(C) of the parent C atom for methyl H, 1.2*U*_iso_(C) for other H.

### Crystal data

**Table:**

C_17_H_16_N_2_O_3_S	*F*_000_ = 688
*M**_r_* = 328.38	*D*_x_ = 1.402 Mg m^−^^3^
Monoclinic, *P*2_1_/*n*	Mo *K*α radiation λ = 0.71073 Å
Hall symbol: -P 2yn	Cell parameters from 11202 reflections
*a* = 9.6018 (3) Å	θ = 2.6–32.1º
*b* = 8.3882 (3) Å	µ = 0.23 mm^−^^1^
*c* = 19.3199 (6) Å	*T* = 100 (2) K
β = 91.393 (4)º	Tablet, colourless
*V* = 1555.60 (9) Å^3^	0.38 × 0.24 × 0.13 mm
*Z* = 4	

### Data collection

**Table:**

Oxford Diffraction Xcalibur S diffractometer	5103 independent reflections
Radiation source: Enhance (Mo) X-ray Source	3676 reflections with *I* > 2σ(*I*)
Monochromator: graphite	*R*_int_ = 0.048
Detector resolution: 16.1057 pixels mm^-1^	θ_max_ = 32.2º
*T* = 100(2) K	θ_min_ = 2.7º
ω scans	*h* = −14→13
Absorption correction: multi-scan(CrysAlis RED; Oxford Diffraction, 2008)	*k* = −12→12
*T*_min_ = 0.943, *T*_max_ = 1.000	*l* = −27→28
31428 measured reflections	

### Refinement

**Table:**

Refinement on *F*^2^	Secondary atom site location: difference Fourier map
Least-squares matrix: full	Hydrogen site location: inferred from neighbouring sites
*R*[*F*^2^ > 2σ(*F*^2^)] = 0.037	H atoms treated by a mixture of independent and constrained refinement
*wR*(*F*^2^) = 0.092	*w* = 1/[σ^2^(*F*_o_^2^) + (0.0546*P*)^2^
*S* = 0.94	(Δ/σ)_max_ < 0.001
5103 reflections	Δρ_max_ = 0.43 e Å^−^^3^
217 parameters	Δρ_min_ = −0.29 e Å^−^^3^
Primary atom site location: structure-invariant direct methods	Extinction correction: none

### Special details

**Table:**

Experimental. CrysAlis RED, Oxford Diffraction Ltd., Version 1.171.32.15 (release 10-01-2008 CrysAlis171 .NET) (compiled Jan 10 2008,16:37:18)
Geometry. All e.s.d.'s (except the e.s.d. in the dihedral angle between two l.s. planes) are estimated using the full covariance matrix. The cell e.s.d.'s are taken into account individually in the estimation of e.s.d.'s in distances, angles and torsion angles; correlations between e.s.d.'s in cell parameters are only used when they are defined by crystal symmetry. An approximate (isotropic) treatment of cell e.s.d.'s is used for estimating e.s.d.'s involving l.s. planes.
Refinement. Refinement of *F*^2^ against ALL reflections. The weighted *R*-factor *wR* and goodness of fit *S* are based on *F*^2^, conventional *R*-factors *R* are based on *F*, with *F* set to zero for negative *F*^2^. The threshold expression of *F*^2^ > σ(*F*^2^) is used only for calculating *R*-factors(gt) *etc*. and is not relevant to the choice of reflections for refinement. *R*-factors based on *F*^2^ are statistically about twice as large as those based on *F*, and *R*- factors based on ALL data will be even larger.

### Fractional atomic coordinates and isotropic or equivalent isotropic displacement parameters (Å^2^)

**Table:**

	*x*	*y*	*z*	*U*_iso_*/*U*_eq_	
S	0.10457 (3)	0.58015 (4)	0.231857 (14)	0.01866 (8)	
O1	0.51573 (9)	0.70720 (10)	0.34044 (4)	0.01821 (18)	
O2	0.50845 (9)	0.81469 (9)	−0.09004 (4)	0.01765 (18)	
O3	0.35152 (9)	0.61785 (9)	−0.10425 (4)	0.01525 (17)	
N1	0.28549 (11)	0.63504 (11)	0.33385 (5)	0.01329 (19)	
H01	0.2098 (15)	0.6256 (15)	0.3555 (7)	0.020 (4)*	
N2	0.37602 (11)	0.65342 (11)	0.22473 (5)	0.0146 (2)	
H02	0.4445 (16)	0.6886 (17)	0.2488 (7)	0.028 (4)*	
C1	0.39896 (12)	0.67621 (12)	0.44636 (5)	0.0126 (2)	
C2	0.48431 (13)	0.78318 (13)	0.48262 (6)	0.0171 (2)	
H2	0.5431	0.8536	0.4582	0.021*	
C3	0.48347 (13)	0.78691 (14)	0.55439 (6)	0.0196 (2)	
H3	0.5392	0.8624	0.5791	0.023*	
C4	0.40113 (13)	0.68031 (14)	0.59006 (6)	0.0190 (2)	
H4	0.4013	0.6822	0.6392	0.023*	
C5	0.31886 (13)	0.57135 (14)	0.55422 (6)	0.0188 (2)	
H5	0.2644	0.4969	0.5789	0.023*	
C6	0.31562 (12)	0.57034 (13)	0.48250 (6)	0.0158 (2)	
H6	0.2567	0.4977	0.4580	0.019*	
C7	0.40746 (12)	0.67435 (12)	0.36961 (5)	0.0131 (2)	
C8	0.26324 (12)	0.62392 (12)	0.26210 (5)	0.0128 (2)	
C9	0.38365 (12)	0.66358 (13)	0.15181 (5)	0.0125 (2)	
C10	0.32131 (12)	0.55175 (13)	0.10741 (5)	0.0142 (2)	
H10	0.2696	0.4656	0.1257	0.017*	
C11	0.33527 (12)	0.56701 (13)	0.03650 (5)	0.0130 (2)	
H11	0.2917	0.4920	0.0061	0.016*	
C12	0.41293 (11)	0.69188 (12)	0.00951 (5)	0.0117 (2)	
C13	0.47771 (12)	0.80015 (13)	0.05441 (5)	0.0139 (2)	
H13	0.5328	0.8837	0.0363	0.017*	
C14	0.46254 (12)	0.78708 (13)	0.12517 (5)	0.0146 (2)	
H14	0.5059	0.8623	0.1555	0.018*	
C15	0.43123 (12)	0.71552 (12)	−0.06585 (5)	0.0123 (2)	
C16	0.36048 (13)	0.63785 (14)	−0.17859 (5)	0.0173 (2)	
H16A	0.4578	0.6239	−0.1932	0.021*	
H16B	0.3287	0.7458	−0.1924	0.021*	
C17	0.26837 (14)	0.51324 (15)	−0.21156 (6)	0.0235 (3)	
H17A	0.3001	0.4072	−0.1968	0.035*	
H17B	0.2728	0.5216	−0.2621	0.035*	
H17C	0.1722	0.5295	−0.1973	0.035*	

### Atomic displacement parameters (Å^2^)

**Table:**

	*U*^11^	*U*^22^	*U*^33^	*U*^12^	*U*^13^	*U*^23^
S	0.01261 (15)	0.03287 (18)	0.01044 (13)	−0.00304 (12)	−0.00088 (10)	0.00021 (11)
O1	0.0142 (4)	0.0261 (5)	0.0143 (4)	−0.0035 (3)	0.0007 (3)	0.0017 (3)
O2	0.0229 (5)	0.0169 (4)	0.0133 (4)	−0.0043 (3)	0.0029 (3)	0.0005 (3)
O3	0.0168 (4)	0.0195 (4)	0.0094 (3)	−0.0034 (3)	−0.0011 (3)	−0.0005 (3)
N1	0.0111 (5)	0.0198 (5)	0.0090 (4)	−0.0013 (4)	0.0007 (3)	0.0001 (3)
N2	0.0140 (5)	0.0205 (5)	0.0092 (4)	−0.0031 (4)	−0.0002 (4)	−0.0004 (3)
C1	0.0131 (6)	0.0139 (5)	0.0106 (5)	0.0026 (4)	−0.0014 (4)	0.0000 (4)
C2	0.0190 (6)	0.0169 (6)	0.0155 (5)	−0.0030 (5)	−0.0005 (4)	−0.0001 (4)
C3	0.0226 (7)	0.0208 (6)	0.0151 (5)	−0.0013 (5)	−0.0037 (5)	−0.0043 (4)
C4	0.0197 (6)	0.0264 (6)	0.0110 (5)	0.0032 (5)	−0.0003 (4)	−0.0005 (4)
C5	0.0168 (6)	0.0254 (6)	0.0141 (5)	−0.0013 (5)	0.0009 (4)	0.0046 (4)
C6	0.0143 (6)	0.0193 (6)	0.0136 (5)	−0.0018 (4)	−0.0023 (4)	0.0003 (4)
C7	0.0142 (6)	0.0128 (5)	0.0121 (5)	0.0007 (4)	−0.0016 (4)	0.0000 (4)
C8	0.0151 (6)	0.0135 (5)	0.0099 (5)	0.0012 (4)	0.0000 (4)	0.0005 (4)
C9	0.0118 (5)	0.0158 (5)	0.0098 (5)	0.0019 (4)	0.0010 (4)	0.0002 (4)
C10	0.0144 (6)	0.0146 (5)	0.0137 (5)	−0.0013 (4)	0.0027 (4)	0.0001 (4)
C11	0.0126 (5)	0.0139 (5)	0.0125 (5)	−0.0002 (4)	0.0008 (4)	−0.0026 (4)
C12	0.0113 (5)	0.0136 (5)	0.0103 (5)	0.0029 (4)	0.0012 (4)	−0.0002 (4)
C13	0.0148 (6)	0.0136 (5)	0.0135 (5)	−0.0011 (4)	0.0017 (4)	0.0004 (4)
C14	0.0147 (6)	0.0171 (5)	0.0121 (5)	−0.0017 (4)	0.0003 (4)	−0.0025 (4)
C15	0.0129 (5)	0.0132 (5)	0.0107 (5)	0.0030 (4)	0.0001 (4)	−0.0007 (4)
C16	0.0224 (6)	0.0216 (6)	0.0078 (5)	−0.0010 (5)	−0.0014 (4)	0.0005 (4)
C17	0.0232 (7)	0.0311 (7)	0.0161 (6)	−0.0049 (5)	−0.0019 (5)	−0.0042 (5)

### Geometric parameters (Å, °)

**Table:**

S—C8	1.6594 (12)	C12—C13	1.3921 (15)
O1—C7	1.2259 (14)	C12—C15	1.4839 (14)
O2—C15	1.2153 (13)	C13—C14	1.3827 (14)
O3—C15	1.3342 (13)	C16—C17	1.5010 (16)
O3—C16	1.4505 (12)	N1—H01	0.852 (15)
N1—C7	1.3850 (14)	N2—H02	0.849 (15)
N1—C8	1.4005 (13)	C2—H2	0.9500
N2—C8	1.3391 (15)	C3—H3	0.9500
N2—C9	1.4151 (13)	C4—H4	0.9500
C1—C2	1.3926 (15)	C5—H5	0.9500
C1—C6	1.3941 (15)	C6—H6	0.9500
C1—C7	1.4871 (14)	C10—H10	0.9500
C2—C3	1.3872 (15)	C11—H11	0.9500
C3—C4	1.3879 (17)	C13—H13	0.9500
C4—C5	1.3829 (17)	C14—H14	0.9500
C5—C6	1.3852 (15)	C16—H16A	0.9900
C9—C14	1.3895 (15)	C16—H16B	0.9900
C9—C10	1.3961 (15)	C17—H17A	0.9800
C10—C11	1.3857 (14)	C17—H17B	0.9800
C11—C12	1.3943 (15)	C17—H17C	0.9800
			
C15—O3—C16	115.63 (9)	C8—N1—H01	111.7 (9)
C7—N1—C8	128.05 (10)	C8—N2—H02	113.3 (10)
C8—N2—C9	127.59 (10)	C9—N2—H02	117.8 (10)
C2—C1—C6	119.77 (10)	C3—C2—H2	120.0
C2—C1—C7	117.54 (10)	C1—C2—H2	120.0
C6—C1—C7	122.59 (10)	C2—C3—H3	120.0
C3—C2—C1	119.97 (11)	C4—C3—H3	120.0
C2—C3—C4	119.94 (11)	C5—C4—H4	119.9
C5—C4—C3	120.17 (10)	C3—C4—H4	119.9
C4—C5—C6	120.24 (11)	C4—C5—H5	119.9
C5—C6—C1	119.85 (10)	C6—C5—H5	119.9
O1—C7—N1	122.71 (10)	C5—C6—H6	120.1
O1—C7—C1	121.58 (10)	C1—C6—H6	120.1
N1—C7—C1	115.70 (10)	C11—C10—H10	120.2
N2—C8—N1	114.55 (10)	C9—C10—H10	120.2
N2—C8—S	126.77 (8)	C10—C11—H11	119.8
N1—C8—S	118.68 (8)	C12—C11—H11	119.8
C14—C9—C10	120.19 (10)	C14—C13—H13	119.7
C14—C9—N2	117.10 (9)	C12—C13—H13	119.7
C10—C9—N2	122.64 (10)	C13—C14—H14	120.1
C11—C10—C9	119.67 (10)	C9—C14—H14	120.1
C10—C11—C12	120.31 (10)	O3—C16—H16A	110.3
C13—C12—C11	119.47 (10)	C17—C16—H16A	110.3
C13—C12—C15	117.54 (10)	O3—C16—H16B	110.3
C11—C12—C15	122.99 (9)	C17—C16—H16B	110.3
C14—C13—C12	120.54 (10)	H16A—C16—H16B	108.6
C13—C14—C9	119.78 (10)	C16—C17—H17A	109.5
O2—C15—O3	123.60 (9)	C16—C17—H17B	109.5
O2—C15—C12	123.83 (10)	H17A—C17—H17B	109.5
O3—C15—C12	112.56 (9)	C16—C17—H17C	109.5
O3—C16—C17	106.93 (9)	H17A—C17—H17C	109.5
C7—N1—H01	119.9 (9)	H17B—C17—H17C	109.5
			
C6—C1—C2—C3	−1.67 (17)	C8—N2—C9—C10	−46.16 (17)
C7—C1—C2—C3	−178.22 (10)	C14—C9—C10—C11	−1.79 (17)
C1—C2—C3—C4	2.21 (18)	N2—C9—C10—C11	−178.66 (10)
C2—C3—C4—C5	−0.63 (18)	C9—C10—C11—C12	1.00 (17)
C3—C4—C5—C6	−1.51 (18)	C10—C11—C12—C13	0.73 (16)
C4—C5—C6—C1	2.04 (18)	C10—C11—C12—C15	−179.62 (10)
C2—C1—C6—C5	−0.45 (17)	C11—C12—C13—C14	−1.70 (17)
C7—C1—C6—C5	175.92 (10)	C15—C12—C13—C14	178.63 (10)
C8—N1—C7—O1	0.74 (17)	C12—C13—C14—C9	0.92 (17)
C8—N1—C7—C1	−179.93 (10)	C10—C9—C14—C13	0.83 (17)
C2—C1—C7—O1	30.47 (15)	N2—C9—C14—C13	177.88 (10)
C6—C1—C7—O1	−145.97 (11)	C16—O3—C15—O2	−0.79 (16)
C2—C1—C7—N1	−148.86 (10)	C16—O3—C15—C12	178.03 (9)
C6—C1—C7—N1	34.69 (15)	C13—C12—C15—O2	6.55 (16)
C9—N2—C8—N1	−174.63 (10)	C11—C12—C15—O2	−173.11 (11)
C9—N2—C8—S	4.22 (17)	C13—C12—C15—O3	−172.27 (10)
C7—N1—C8—N2	0.99 (16)	C11—C12—C15—O3	8.07 (15)
C7—N1—C8—S	−177.97 (9)	C15—O3—C16—C17	177.67 (10)
C8—N2—C9—C14	136.88 (12)		

### Hydrogen-bond geometry (Å, °)

**Table:**

*D*—H···*A*	*D*—H	H···*A*	*D*···*A*	*D*—H···*A*
N2—H02···O1	0.85 (2)	1.89 (2)	2.618 (1)	143 (1)
N1—H01···O2^i^	0.85 (2)	2.28 (2)	3.099 (1)	162 (1)
C3—H3···S^ii^	0.95	3.04	3.763 (1)	134
C17—H17C···S^iii^	0.98	2.88	3.677 (1)	140

Symmetry codes: (i) *x*−1/2, −*y*+3/2, *z*+1/2; (ii) *x*+1/2, −*y*+3/2, *z*+1/2; (iii) −*x*, −*y*+1, −*z*.
